# Functions of the Spinal Cord

**Published:** 1847-10

**Authors:** 


*Functions of the Spinal Cord.*—Some curious results have been afforded by an ingenious set of experiments performed by M. Brown-Séquard for ascertaining the degree of motor power left in the spinal cord after separation from the brain, compared with the power which can be exerted while its connection with the brain is left uninjured. The process which he employed for ascertaining this consisted in suspending from the extremity of one of the posterior limbs of a frog a light weight, sufficient to keep the limb stretched; and then pinching one of the toes, whereupon flexion of the limb with its attached weight was excited. By increasing the suspended weight until it amounted to more than the animal by its attempts at flexion of the limb could move, a tolerable correct estimate might be formed of the degree of voluntary motor power the frog was capable of exerting. Having determined this, he divided the spinal cord below the origin of the second pair of nerves, and then observed the effect which such division had on the motor power of the limb. Immediately after the division, he finds that sometimes the motor power is for the time quite lost, although there generally remains about one-quarter or one-third of what there was before the operation; at other times there is left about one half, or even, though very rarely, as much as two-thirds. A frog, for example, which before the operation could raise a weight amounting to 60 grammes, would not be able immediately after it, to raise any weight at all, or one amounting to 10, 20, 30, or 40 grammes, but not so much as 60 grammes. In five minutes after the operation the motor power is sensibly increased, and usually amounts to one-third, one half, or even two-thirds of its original amount. In fifteen minutes it has increased still more; and in from twenty to twenty-five minutes generally amounts to what it was before the operation. In an hour after the operation its original amount is sometimes doubled, and in two or three hours it is often trebled. Arrived at this degree, the motor power appears to cease from further increase, though sometimes it does not attain this, which may be regarded as its maximum, until twenty-four hours after the operation; and sometimes it is two or three days in attaining it: but in all such cases, the increase is very slow after the first few hours succeeding the operation. When once attained, the maximum motor force remains stationary for five, ten, fifteen, or twenty days, and then gradually diminishes. If the frog survives the operation for some months, the motor force is reduced lower than it was before the operation. But probably if the animals were nourished, and if movements of the hind limbs were frequently excited, this diminution would not take place,—or, at any rate, not to so great an extent.—*London Med. Gaz., from Comptes Rendus.*

